# Radiation-induced kidney injury

**DOI:** 10.12861/jrip.2012.17

**Published:** 2012-09-01

**Authors:** Milad Baradaran-Ghahfarokhi

**Affiliations:** Medical Physics and Medical Engineering Department and Medical Student’s Research Center, School of Medicine, Isfahan University of Medical Sciences, Isfahan, Iran

**Keywords:** Radiation therapy, Kidney injury, Radiation toxicity

**Implication for health policy/practice/research/medical education:**

Radiotherapy with or without chemotherapy for pelvic malignancies such as gastrointestinal cancers, gynecologic cancers, lymphomas, and sarcomas of the upper abdomen and during total body irradiation may result in radiation-induced kidney injury. The incidence of clinical radiation nephropathy has increased with the use of total-body irradiation in preparation for bone marrow transplantation. Radiation nephropathy usually manifests as proteinuria, hypertension and impairment in urine concentration. The precise pathogenic mechanisms and/or mediators involved in radiation nephropathy remain under active investigation. However, radiation nephropathy is no longer viewed as inevitable, progressive, and untreatable.

The kidneys are vitally important, responsible for producing erythropoietin to stimulate red blood cell production, filtering waste metabolites and electrolytes from the blood, and modulating blood pressure by fluid/electrolyte balance (1). In pelvis radiation therapy (RT), the pronounced radiosensitivity of renal tissue limits the total radiotherapeutic dose that can be applied safely to treatment volumes that include the kidneys (2).

Radiotherapy with or without chemotherapy for pelvic malignancies such as gastrointestinal cancers, gynecologic cancers, lymphomas, and sarcomas of the upper abdomen and during total body irradiation (TBI) may result in radiation-induced kidney injury, especially radiation nephropathy (RN) (1,3,4). The incidence of clinical RN has increased with the use of TBI in preparation for bone marrow transplantation (BMT) and as a consequence of radionuclide therapies. BMT nephropathy usually develops very slowly, over a period of several years, and manifests as proteinuria, hypertension and impairment in urine concentration (4,5).

It is possible that radiation nephropathy could occur after a nuclear accident or because of nuclear terrorism (6,7). Exposures that would cause this would have to be in the 5 to 10 Gy range. “Doses less than 5 Gy would not materially affect the kidneys, whereas doses greater than 10 Gy would cause rapid gastrointestinal death” (8-10).

In RN, as in normal tissue radiation injury in general, it is not possible to predict which subjects will develop the complication. However, considering the threshold dose, sufficient ionizing radiation injures most or all components of the kidney. Glomerular injury is chronologically first, and involves at least its endothelium and mesangium, with evolution to glomerular scarring due to thrombotic microangiopathy. Expression of tubular injury appears to occur somewhat later, even if it is set in motion at the same time as the glomerular injury. Oxidative injury to the glomeruli, could play a mechanistic role. Denuded tubules could allow interstitial entry to mediators that escape from injured glomeruli. Local mediator expression, such as TGFβ1 or activation of renin-angiotensin system could be key in creating tubulointerstitial scarring. Moreover, there are some rare syndromes of radiation sensitivity such as ataxia telangiectasia, but these are not clinically frequent (4). Management of radiation nephropathy includes attention to control of blood pressure and the use of angiotensin-converting-enzyme inhibitors (ACE inhibitors) or angiotensin (AII) receptor blockers (11,12).

## Conclusion

Radiation-induced kidney injury involves complex and dynamic interactions between glomerular, tubular, and interstitial cells. Although the precise pathogenic mechanisms and/or mediators involved in radiation nephropathy remain under active investigation. Radiation nephropathy is no longer viewed as inevitable, progressive, and untreatable.

## Author’s contribution

MBG is the single author of the manuscript.

## Conflict of interests

The author declared no competing interests.

## Ethical considerations

Ethical issues (including plagiarism, data fabrication, double publication) have been completely observed by the author.

## Funding/Support

None.
